# Noninvasive measurement of ^13^Carbon turnover for evaluation of porcine renal grafts during ex vivo machine perfusion

**DOI:** 10.1038/s43856-023-00422-6

**Published:** 2023-12-21

**Authors:** Thomas Minor, Laura Malkus, Hristo Zlatev, Bastian Lüer, Charlotte von Horn

**Affiliations:** Surgical Research Department, University Hospital Essen, Hufelandstr. 55, 45147 Essen, Germany

**Keywords:** Kidney, Biomarkers

## Abstract

**Background:**

Kidney transplantation suffers from a shortage of donor organs. Despite this, a lot of grafts are discarded due to inadequate quality. As many kidneys are afflicted by transient filtration failure early after preservation, classical renal function tests are not applicable to differentiate between prospective recovery or continuing deficit of renal function.

**Methods:**

Using normothermic machine perfusion as a platform for pre-implantation evaluation of the graft, we present a novel evaluative approach based on the metabolic turnover of ^13^C-acetate during isolated perfusion. After injection of the tracer, ^13^CO_2_ as a metabolic end-product can be quantified by high-precision laser-based spectroscopy in the gas outflow of the oxygenator. Three groups of porcine kidneys with graduated ischemic injury were investigated.

**Results:**

This quantitative approach is able to discriminate acceptable quality kidneys, most likely to recover within days from poor kidney grafts that are unlikely to regain notable glomerular function with high discriminatory power (area under the ROC curve 0.91; *P* < 0.001 By contrast, conventional renal function tests are rather ineffective under these circumstances.

**Conclusions:**

This assessment method offers the potential to quantitatively assess donor kidney quality using a measurable output, salvaging donors that would otherwise have been discarded.

## Plain language summary

Kidney transplant surgery continues to face donor shortages. One consideration to tackle the shortage is to improve the way in which donor organ quality is assessed, so that fewer organs may be discarded. Here, we develop and test a non-invasive quantitative method that can measure the biochemical reaction that correlates to the viability of isolated kidneys using pig kidneys as a model system. We find the method could discriminate donor kidneys of good, intermediate, or poor quality with a discriminatory power superior to all other conventional parameters. Although the application needs to be tested in human kidney donors, it offers the potential to improve evaluation criteria for kidney grafts for transplantation.

## Abstract (web summary)

Minor et al. present and evaluate a quantitative approach to measuring metabolic turnover of ^13^C-acetate during isolated perfusion to ascertain the quality of porcine donor kidneys. This approach effectively discriminates varying degrees of organ graft quality, where conventional renal function tests are ineffective.

## Introduction

Organ donation is a highly debated topic all over the world due to several ethical, social, and medical aspects. The steadily increasing demand for donor organs forces politics and science to find new strategies for enlargement of the donor pool and availability of grafts for life-saving transplantation^1^. It is not unlikely that the limits of acceptance criteria for the use of donor grafts will expand in the future and this will represent a valuable advance to enlarge the total number of organs available for transplantation.

However, these ‘less than optimal’ grafts are often conflicted with a reduced functional reserve and, hence, are less resilient against preservation and reperfusion injury. In line with the attempt to push the limits of acceptance criteria, tools to support the surgeon with an appropriate estimation of the quality of organ grafts are of crucial importance and yet basically underdeveloped. The safe and successful extension of donor organ criteria has to be associated with evaluative measures, helping to decide whether a marginal graft should actually be used for transplantation or rather be discarded.

A plethora of biomarkers, obtained during isolated machine perfusion, has already been tried to evaluate morphological injury to the graft but correlations with ulterior function of transplanted kidneys were generally insufficient to be used in isolation for discard decisions^2,3^.

Altogether, biological indicators of -potentially reversible- morphological tissue disturbances may be less pertinent to predict renal recovery after transplantation than a functional evaluation of the isolated perfused kidney graft. Thus, normothermic machine perfusion evolves as promising tool for graft evaluation prior to transplantation. At present, it seems fairly easy to disclose differences upon isolated machine perfusion between well-functioning kidneys and poor kidneys, that suffer from a delayed resumption of renal filtration function^4,5^.

However, clinical experience has shown that glomerular filtration can initially be perturbated in a large number of nonetheless acceptable donor kidneys that frequently regain full renal function during the first days after transplantation^6,7^. Therefore, deprivation of glomerular filtration upon machine perfusion prior to engraftment does not necessarily preclude a successful outcome after transplantation and methods to discern between transient but reversible renal dysfunction and irreversible non-function of a kidney graft would be of imperative interest^8^.

In this article, we propose a novel approach to discriminate between good, acceptable, and poor renal grafts based on the metabolic turnover of ^13^C-acetate of the isolated perfused kidney.

After injection of ^13^C-acetate and metabolization of the tracer in the tricarboxylic acid cycle, the released ^13^CO_2_ is transported by the venous effluent to the oxygenator, where it can be quantified by a laser-based spectroscopy in the gas outflow using a high precision detector that simultaneously measures temperature, flow, and pressure. Here, we show that the production rate of ^13^CO_2_ can discriminate (area under the ROC curve 0.91; P < 0.001) between acceptable kidneys, most likely to recover within days and poor kidney grafts, that are likely never to restore notable glomerular function again.

## Methods

### Ethics declarations

The study complies with all relevant ethical regulations for animal testing and research. Experiments were carried out only on isolated kidneys that were retrieved from dead female pigs (German Landrace, age between 12 and 14 weeks) after euthanasia by i. v. injection of potassium chloride, in deep anesthesia. The procedure of euthanasia for organ retrieval according to § 4 Abs. 3, TSG (German Legislation on animal protection) has been approved by the responsible authority LANUV (Landesamt für Natur, Umwelt, und Verbraucherschutz NRW, Germany).

The principles of laboratory animal care (NIH publication no. 85-23, revised 1985) were followed. No Heparin was given at any time.

### Experimental groups

Based on our own experiences and reports from the literature on experimental porcine kidney transplantation, three different groups of investigation were created that not only represent gradual increases in renal injury, but were matched to established graft outcome data after renal transplantation in the pig. Thus, good, acceptable (fair), and poor kidney grafts were investigated according to different times of warm ischemic injury prior to cold storage, that are reported to result in 100% initial graft function, high rates of delayed graft function but ultimate recovery and usual primary non-function, resp^9,29,30^.

Kidneys were randomly assigned to one of the following groups:

Good kidney grafts (*n* = 13): Kidneys were removed ~5 min after cardiac standstill of the donor animal. According to own previous experiences, such kidneys do perform virtually identical to kidneys retrieved during intact circulation.

Acceptable kidney grafts (*n* = 13): The group is intended to represent fair donor organs that might be usable in great majority but usually do already show a reduced initial graft function. These kidneys were removed only 30 min after cardiac standstill of the donor animal. In porcine kidney transplantation, 30 min of warm ischemia prior to cold storage is reported to be afflicted with a high rate of delayed graft function^31^, but usually associated with 100% ulterior graft survival^9^.

Critical kidney grafts (*n* = 13): Aiming to create a group of poor donor organs, these kidneys were removed only 60 min after cardiac standstill of the donor as has been reported to result in 100 % nonfunctioning porcine grafts^30,32^.

Even if Heparin would have been administered prior to induction of circulatory standstill, which might notably improve the outcome as compared to non-anticoagulated settings, 3-month survival after 60 min of warm ischemia is less than 50 %^33^.

In line with this, a warm ischemic time of less than 45 min has recently been postulated as acceptance criterion for human kidney donation after cardiac standstill of the donor, according to Maastricht III criteria in a comparable setting^10^.

In all kidneys, the renal artery was cannulated and the kidneys were flushed by 100 cm gravity with 100 ml of HTK solution (Köhler Chemie, Bensheim, Germany) on the back-table at 4 °C and preserved in a beaker filled with HTK solution kept at 4 °C by the help of a cryo-thermostat.

### Normothermic machine perfusion

After 18 h of static storage, kidneys from all groups were put on a machine perfusion circuit and subjected to an end-ischemic machine perfusion of 3 h duration.

According to previous experiences to mitigate tissue damage by abrupt temperature increase (rewarming injury)^34^, the temperature of the perfusate was slowly elevated from initially 8 °C up to normothermia during the first 30 min with concomitant adjustment of the perfusion pressure from 30 mmHg to 75 mmHg. Perfusate consisted of Aqix RS I solution and was supplemented with bovine serum albumin (40 g/l), sodium bicarbonate 8.4 % (25 ml), and dexamethasone (4 mg). Creatinine was also added at 6 mg/100 ml to allow for measurement of glomerular filtration rate during machine perfusion^35^.

Perfusion pressure was regulated by a servo-controlled pump that adjusted the renal flow according to the feedback obtained by a connected pressure transducer in the arterial inflow line. Oxygenation was provided by an interposed membrane oxygenator fed at a constant flow rate of 0.6 l/min.

### ^13^C-Acetate turnover test

During normothermic end-ischemic machine perfusion, metabolic measurements on citrate cycle throughput were performed by ^13^C detection in the gas outlet of the oxygenator after bolus injection of 25 mg of ^13^C-Acetate.

**Fig. 1:**
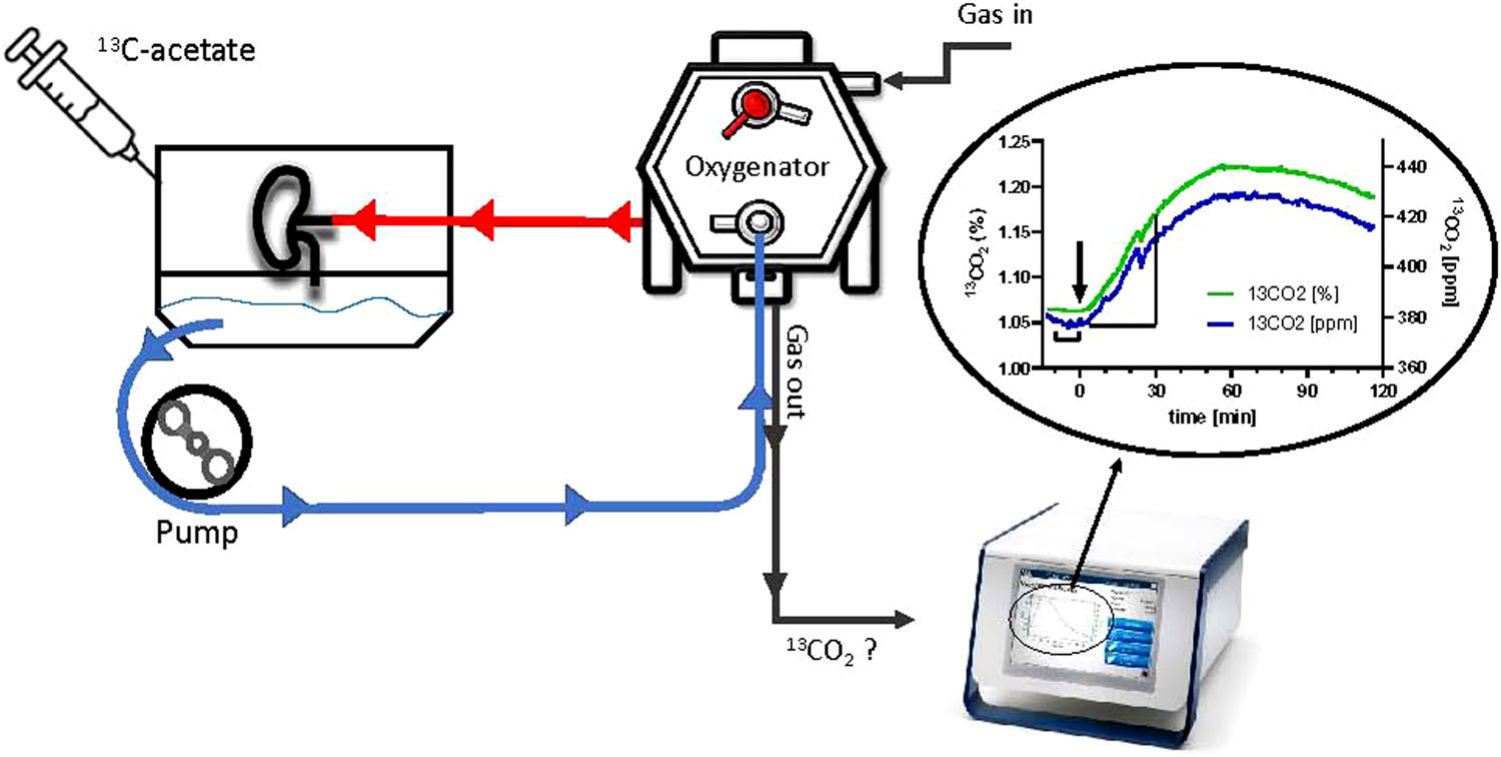
Simplified schematic representation of the ^13^C-acetate test during normothermic machine perfusion of kidney grafts. After obtaining steady state conditions during graft perfusion the baseline values for ^13^CO_2_ and the ^13^CO_2_/^12^CO_2_ ratio are recorded before application of the tracer. Injection of 25 mg of ^13^C-acetate will result in its metabolization in the renal tissue by the TCA-cycle yielding ^13^CO_2_ that is released in the venous outflow and subsequently excreted by the oxygenator in the gas outflow line. Thus, it can be detected by a notable rise of the ^13^CO_2_ recording over the baseline level. The amount of substance detected during the first 30 min after injection of the tracer is taken as a readout of the respective metabolic activity of the kidney graft.

Detection was executed using the novel high-precision ^13^CORlab device (ArgosMED, Karlsruhe, Germany). The device was connected to the gas-outflow of the oxygenator and directly detected the concentration of ^12^CO_2_ as well as^13^CO_2_ molecules during the gas transit through the detector, using laser-based spectroscopy along with the measurement of flow, pressure, and temperature of the gas (cf. Fig. 1).

The set-up enables the calculation of the exact amount of substance of ^13^CO_2_ at a given time point with a sampling rate of 1/s. The metabolized amount of ^13^CO_2_ corresponds to the difference of the detected concentration of ^13^CO_2_ and the concentration of ^13^CO_2_ at baseline conditions (i.e. before application of the test metabolite). The amount of substance can be derived by multiplying the metabolized concentration of ^13^CO_2_ with the total amount of substance of the analyzed gas. The latter can be determined as total volume per time divided by the molar gas volume at standard conditions.

1 $${\eta }^{13}{{{{\rm{CO}}}}}_{2}(t)=[{{{{\rm{C}}}}}^{13}{{{{\rm{CO}}}}}_{2}({{{\rm{t}}}})-{{{{\rm{C}}}}}^{13}{{{{\rm{CO}}}}}_{2}({{{\rm{baseline}}}})]\times \eta \,{{{\rm{tot}}}}$$

2 $$=[{{{{\rm{C}}}}}^{13}{{{{\rm{CO}}}}}_{2}({{{\rm{t}}}})-{{{{\rm{C}}}}}^{13}{{{{\rm{CO}}}}}_{2}({{{\rm{baseline}}}})]\times {{{\rm{V}}}}({{{\rm{t}}}})/{{{\rm{V}}}}({{{\rm{st}}}})$$

η: Amount of substanceC ^13^CO_2_: Concentration of ^13^CO_2_ at given time pointC ^13^CO_2_: Concentration of ^13^CO_2_ at baselineV(t): Total gas volume per given timeV(st): Molar gas volume at standard conditions = 22.4 L/mol)

Thus, the metabolic rate can be determined during the linearly rising part of the slope of measured amount of substance over time and is calculated in our study as µmol/min based on a measurement interval of 30 min.

### Conventional markers of graft integrity

Renal perfusate flow was detected by using an electromagnetic sensor inserted into the arterial inflow line. The ureter was cannulated and the urine collected during the entire perfusion period. Lactate levels in the perfusate were measured in an acid-base laboratory (ABL800 flex, Radiometer, Copenhagen, Denmark). Creatinine was measured in a routine fashion by reflectance photometry on an Element RC3X point of care unit (scil animal care company, Viernheim, Germany). Urinary alpha glutathione S transferase (GST) was quantified using a porcine ELISA test according to the manufacturer’s instruction (My biosource, San Diego, USA).

### Statistics and reproducibility

The hypothesis that the measurement of ^13^C-acetate metabolism would be valuable to discriminate between the predefined levels of kidney quality should be tested by establishing a respective receiver operating curves (ROC), defining the area under the curve for accurate discrimination by ^13^C-acetate metabolism measurements between good and acceptable or acceptable and poor grafts. Thereby, an area under the curve of 0.8 or better should be considered as a significant benefit for future use and further investigation of the parameter. The rate of type 2 error has been defined as ß = 0.2. The level of significance for the type 1 error is set to p < 0.05. From these assumptions, it follows that the detection of a discriminative significance compared to the null hypothesis (Area under the ROC curve = 0.5) requires a sample size of *n* = 13 per group (MedCalc® Statistical Software version 20.009 (MedCalc Software Ltd, Ostend, Belgium; https://www.medcalc.org; 2021).

All values were expressed as means ± SD unless otherwise indicated. Differences between groups were tested by one-way ANOVA and parametric comparison of the means using Tukey-Kramer test. Statistical significance was set at P < 0.05. ROC curve data were calculated using Prism 9.01 (GraphPad software Inc., San Diego, CA, USA).

### Reporting summary

Further information on research design is available in the Nature Portfolio Reporting Summary linked to this article.

## Results

### Baseline characteristics

Thirty-nine porcine kidneys were subjected to 5, 30, or 60 min of warm ischemic injury prior to cold storage, representing good, acceptable, and poor renal grafts, respectively, according to previous experiences in porcine kidney transplantation^9,10^. Organ weight averaged 71.6 ± 16.5 g vs 71.4 ± 12.6 g vs 75.6 ± 9.1 g in good, acceptable, and poor grafts with no significant differences between the groups.

Upon normothermic machine perfusion (NMP) all kidneys reached steady-state conditions within the first 60 min on the machine and a stable baseline of the ratio of ^13^CO_2_/^12^CO_2_ could be registered in the outflow line coming from the oxygenator.

### Functional parameters upon ex vivo machine perfusion

After injection of the ^13^C -acetate into the perfusate, a quick increase in the ^13^CO_2_ detected at the oxygenator outflow was indicative for renal metabolism of the tracer and allowed for the calculation of the metabolized amount of substance during a given time.

**Fig. 2:**
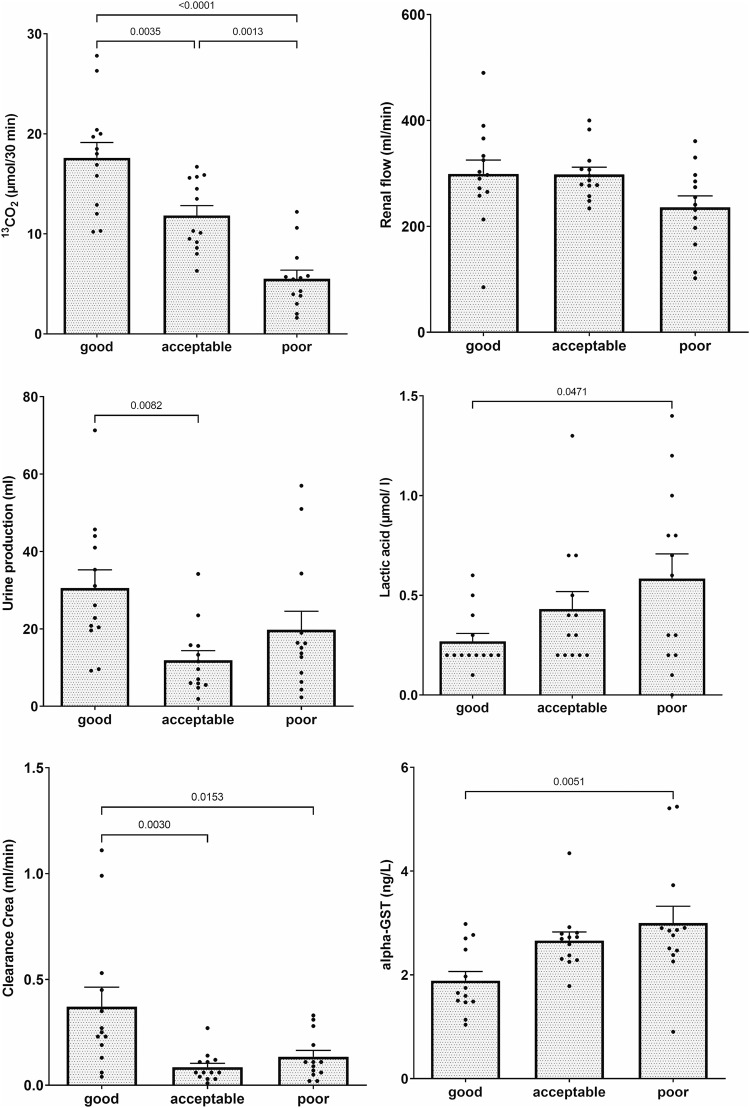
Influence of graft quality on functional markers upon machine perfusion. Differences between functional markers of kidney graft integrity obtained during normothermic machine perfusion of good, acceptable, and poor kidney grafts (5, 30, or 60 min of warm ischemic injury prior to cold storage), respectively. Data are given a mean ± SD and individual values of *n* = 13 per group; p values (if significant) by one-way ANOVA and Tukey-Kramer test.

The amount of ^13^CO_2_ production after injection of ^13^C-acetate was sensibly affected by the degree of renal graft quality and differed significantly between good and acceptable organs as well as between acceptable and poor kidney grafts (cf. Fig. 2).

For comparison, a variety of other conventional functional parameters frequently used for graft evaluation upon NMP have been evaluated in parallel and were also and stratified for good acceptable and poor kidney quality according to the experimental protocol.

No significant differences between any of the groups could be evidenced for renal flow, corresponding to renal vascular resistance in this model of constant pressure perfusion.

Cumulative urine production during NMP did show differences between good and acceptable grafts but did not allow for discrimination of poor grafts from either good or acceptable kidneys.

Perfusate levels of lactate exhibited a consistent tendency towards higher values in more injured renal grafts and disclosed a significant difference between good and poor grafts. However, no actual discrimination between acceptable and poor kidneys was possible.

Renal clearance of creatinine was also measured as a central parameter of kidney function.

It was seen that good grafts actually had a notably and significantly higher clearance than the other two groups but renal clearance during NMP was already severely affected even in acceptable kidneys with no further deterioration disclosed in poor kidney grafts.

Urinary concentrations of alpha glutathione-S-transferase (GST) have been determined for being a most commonly evaluated biomarker^11^ for kidney injury. In our model, we could see a significant intergroup difference between good and poor renal grafts but values in acceptable kidneys did not significantly differ from either good or poor grafts.

### Receiver operator characteristics

**Fig. 3:**
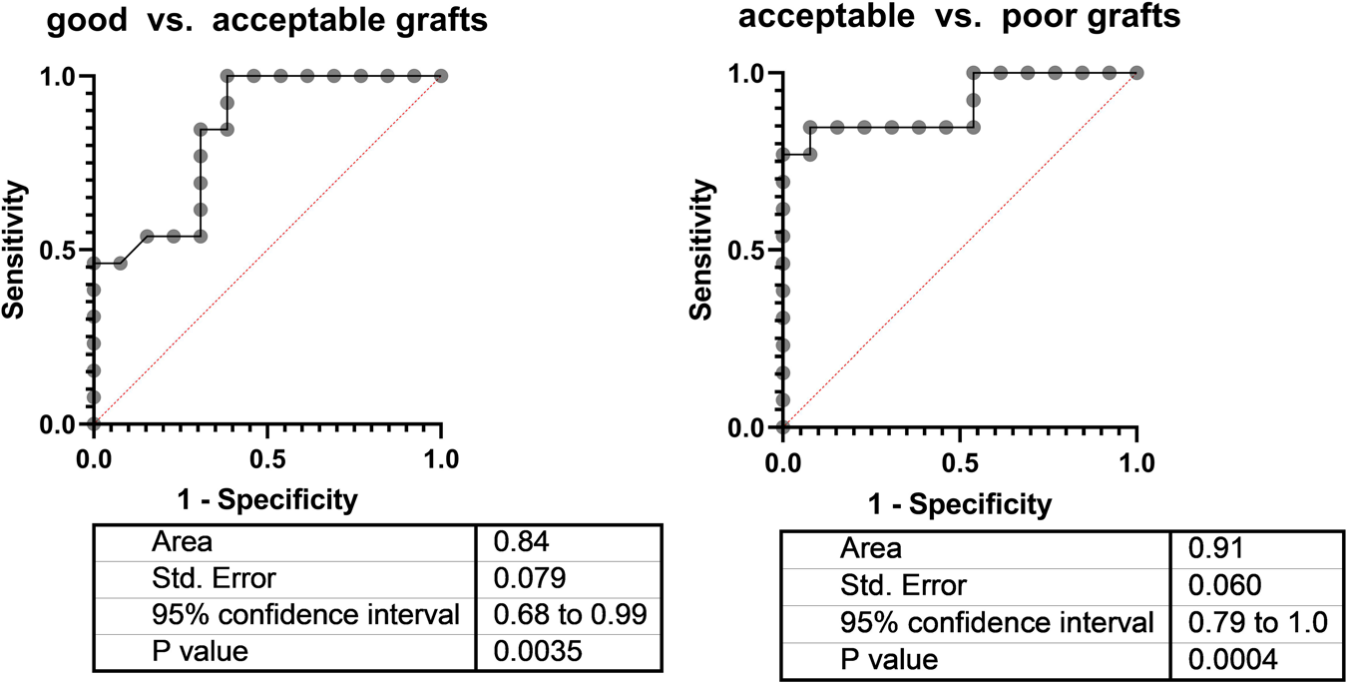
Discriminative value of ^13^C-acetate metabolization. Receiver-operating characteristics curves illustrate the accuracy for ^13^C-acetate metabolization observed during NMP to allow for discrimination between kidneys of different quality. Data were calculated from *n* = 13 per group.

The receiver operating characteristics curves of ^13^C-acetate metabolization for discrimination between good and acceptable grafts as well as between acceptable and poor grafts are shown in Fig. 3. It is seen that the parameter not only allows for a rather acceptable discrimination between good and acceptable grafts (area under the curve 0.84) but displays an even better separation of acceptable from poor kidneys. The area under the curve (AUC) being 0.91, this result thus fulfils the primary goal of the present investigation establish a new parameter yielding an AUC of > 0.8.

Tables 1 and 2 compare the areas under the receiver operator characteristics curves and the 95% confidence intervals for the ^13^C-acetate metabolization with all of the other evaluative parameters under investigation.

**Table 1:** Discrimination between good and acceptable kidney grafts.

Parameter	Area (95% CI)	*P* value
^13^C-acetate	0.84 (0.68–0.99)	0.0035
Renal flow	0.53 (0.30–0.76)	0.7779
Urine production	0.86 (0.72–1.0)	0.0018
Lactic acid	0.68 (0.48–0.89)	0.1119
Clearance	0.86 (0.72–1.0)	0.0016
alpha GST	0.80 (0.62–0.98)	0.0096

Area under the receiver operator curve (ROC), 95% confidence interval (CI), and level of significance to discriminate between good and acceptable kidney grafts by various functional parameters obtained upon normothermic machine perfusion (13C-acetate: production of 13 CO2 after injection of 13C-acetate).

**Table 2:** Discrimination between acceptable and poor kidney grafts.

Parameter	Area (95% CI)	*P* value
^13^C-acetate	0.91 (0.79–1.0)	0.0004
Renal flow	0.75 (0.56–0.95)	0.0293
Urine production	0.66 (0.44–0.87)	0.1742
Lactic acid	0.59 (0.35–0.82)	0.4571
Clearance	0.63 (0.41–0.85)	0.2702
alpha GST	0.64 (0.42–0.86)	0.2282

Area under the receiver operator curve (ROC), 95% confidence interval (CI), and level of significance to discriminate between acceptable and poor kidney grafts by various functional parameters obtained upon normothermic machine perfusion (13C-acetate: production of 13CO2 after injection of 13C-acetate).

Most of the parameters were able to rather well separate the good kidneys from the acceptable with best values and AUC > 0.8 for the clearance of creatinine, urine production and the ^13^C-acetate metabolization (Table 1).

Of note, all of these parameters, except for the ^13^C-acetate metabolization did fail to yield significant results, when poor grafts had to be separated from still acceptable ones (cf. Table 2).

In this scenario, ^13^C-acetate metabolization proved to be highly significant with an AUC of 0.91.

The only other parameter reaching significance to discriminate between poor and acceptable kidneys level was total renal perfusate flow with an AUC of 0.75.

## Discussion

This study introduces an easy and non-invasive method for the evaluation of the tri-carboxylic acid (TCA) cycle activity in renal organ grafts upon isolated machine perfusion.

Exogenous acetate is rapidly extracted by renal cells^12^. Once inside the cell, it will be metabolized via acetyl-CoA synthetase to acetyl-CoA which enters the TCA cycle in proportion to the oxidative rate of the cycle^13^. The radioactive isotope ^11^C-acetate has already been used as a tracer in positron emission tomography (PET) imaging to study myocardial blood flow and oxidative metabolism where the clearance of the tracer was considered a direct reflection of TCA activity^14^.

Different from the situation in the whole body, the set-up of isolated organ perfusion makes sure, that all of the applied tracer is taken to the kidney and only metabolized by the kidney.

Every released molecule of ^13^CO_2_ detected in the gas outflow can be attributed to renal metabolization of ^13^C-acetate and there is no need to specifically attribute the tracer as being pertinent to the kidney.

Using the non-radioactive ^13^C-isotope and a portable high-precision detector, the method described in this article allows for a most versatile determination of renal TCA activity prior to engraftment.

The depression of glomerular filtration early after ischemia may often represent a temporary failure that does not necessarily preclude later resumption of renal function^15^. Only a prolonged duration (e.g. up to 2 weeks) of poor function could be considered as predictor of compromised long-term outcome^6,16^.

Of note, the rate of ^13^C-acetate metabolization turned out to be much more decisive than other parameters under investigation when it came to separating the poor grafts from the acceptable kidneys.

This would be of clinical importance in so far as good kidneys as well as fair, but acceptable renal grafts will actually be transplanted, the challenge lying in the recognition and discard of those grafts, that are not likely to recover after engraftment.

Acetate counts among the most used circulating metabolites taken up by the kidney^17^ and directly drives the TCA cycle^18^. Nonetheless, other metabolites might serve as well as a tracer for the ^13^C gas detection upon ex vivo machine perfusion. Lactate, for instance, is also a major TCA substrate and a possible tracer for metabolic activity. However, it has been shown to accumulate upon perfusion of marginal or discarded kidney grafts^19–21^. High levels of lactate may result in impaired reduction of pyruvate to lactate by product inhibition of lactate dehydrogenase (the activity of which is already compromised by ischemia^22^) and foster cellular oxidation-reduction stress^23^. Thus, the deliberate addition of lactate to the system might be seen controversial in cases of questionable renal quality.

Pyruvate on the other hand has been shown to deplete during ischemia and early reperfusion and ^13^C-pyruvate might be an alternative tracer for the evaluation of renal intermediate metabolism.

In order to support surgical decision making on discard or acceptance of a questionable graft, a diagnostic parameter needs to be available *ad hoc* prior to engraftment. In this regard, the real-time acetate turnover test recommends itself as more practicable than other objective parameters recently proposed for renal graft evaluation.

Specific biomarkers like alpha-GST were often assessed only in retrospect, using time-consuming ELISA test kits and are hence of questionable value in the concrete pre-transplant situation.

Moreover, many investigations on pre-transplant renal graft evaluation have focused on the differentiation between only two groups of graft quality. Clinical studies are confined to the comparison of those grafts that were actually transplanted and do thus usually not include kidneys of questionable quality that had (perhaps unnecessarily) been discarded for safety reasons. As in our study, perfusate concentrations of alpha-GST, as the most commonly evaluated biomarker, have shown a moderate predictive value in larger clinical series, where good kidneys showed lower values than accepted kidneys with rather fair function after transplantation^2,3^. To date, it is generally accepted that perfusate biomarker measurements should not lead to kidney discard^2,24^, which is quite in line with the results of the present study, where kidneys of poor quality could not be differentiated from acceptable grafts by this parameter.

The only ad hoc parameter, apart from the acetate turnover test, that showed off to be helpful in discriminating acceptable from poor kidneys in our study had been the renal perfusate flow.

This finding may relate to the fact that vascular dysfunction represents a prominent exponent of ischemia reperfusion injury in the kidney^25–27^ and reduced renal perfusion favors secondary tissue hypoxia^28^.

Moreover, it corroborates earlier observations where blood flow during MP has already been identified as one criterion when scoring kidneys for transplantation^15^.

One limitation of our model may be seen in the fact that no individual correlation with renal function after transplantation had been established. Ethical considerations and local regulations strongly disfavor survival experiments as the primary approach for hypothesis testing.

The focus of our experiments was thus set on the discriminative power of the ^13^C turnover test in comparison with conventional renal function tests to separate the fairly injured kidneys from the severely injured ones. The acetate turnover values obtained in or study are hence to be seen as only indirectly correlated to renal outcome prognosis, based on experiences with collectives of kidney transplants subjected to identical degrees of ischemic injury. Other than that, an instantaneous transfer into clinical decision making on graft discard or acceptance is problematic, mainly because metabolic benchmarks in humans may differ from those, we have seen in porcine grafts.

Therefore, the presented method is not yet proposed as a stand-alone tool for deciding of acceptance or rejection of a questionable renal graft. Nonetheless, the ^13^C turnover test has shown a significant discriminative power, especially in the lower range of renal graft quality. The test was superior to a variety of established parameters and appears to have the potential of a consistent and valuable adjunct in the toolbox for pre-transplant evaluation of questionable renal grafts.

Final validation of this tool can only be performed upon long-term follow-up in clinical exploration, also including grafts suffering from reduced resilience due to reasons other than ischemic injury like higher age or accompanying diseases of the donor. The explorative translation into clinical practice might provide an objective help to minimize unnecessary discard of marginal kidneys.

### Supplementary information

Supplementary Data 1

Reporting Summary

## Data Availability

Source data underlying Figs. 2 and 3 can be found in Supplementary Data 1. All other data are available from the authors upon reasonable request.

## References

[1] Saidi RF, Hejazii, Kenari SK (2014). Challenges of organ shortage for transplantation: solutions and opportunities. Int. J. Organ Transplant. Med..

[2] Moers C (2010). The value of machine perfusion perfusate biomarkers for predicting kidney transplant outcome. Transplantation.

[3] Parikh CR (2016). Associations of perfusate biomarkers and pump parameters with delayed graft function and deceased donor kidney allograft function. Am. J. Transplant..

[4] Minor T, Sutschet K, Witzke O, Paul A, Gallinat A (2016). Prediction of renal function upon reperfusion by ex situ controlled oxygenated rewarming. Eur. J. Clin. Invest..

[5] Hunter, JP et al. Assessment of mitochondrial function and oxygen consumption measured during ex vivo normothermic machine perfusion of injured pig kidneys helps to monitor organ viability. *Transplant Int.* **35** (2022). 10.3389/ti.2022.10420 PMC9194576 35711321

[6] Phillips, BL et al. Effect of delayed graft function on longer-term outcomes after kidney transplantation from donation after circulatory death donors in the United Kingdom: a national cohort study. *Am. J. Transplant.* **21**, 3346–3355 (2021). 10.1111/ajt.16574 33756062

[7] Bahl D, Haddad Z, Datoo A, Qazi YA (2019). Delayed graft function in kidney transplantation. Curr. Opin. Organ Transplant..

[8] Jochmans I, Lerut E, van PJ, Monbaliu D, Pirenne J (2011). Circulating AST, H-FABP, and NGAL are early and accurate biomarkers of graft injury and dysfunction in a preclinical model of kidney transplantation. Ann. Surg..

[9] Schreinemachers MCJM (2009). Improved renal function of warm ischemically damaged kidneys using polysol. Transplant. Proc..

[10] Dauger S (2022). Organ donation by Maastricht-III pediatric patients: recommendations of the Groupe Francophone de Réanimation et Urgences Pédiatriques (GFRUP) and Association des Anesthésistes Réanimateurs Pédiatriques d’Expression Française (ADARPEF). Part II: specific organizational and technical considerations. Arch. Pédiatr..

[11] De Beule J, Jochmans I. Kidney perfusion as an organ quality assessment tool-are we counting our chickens before they have hatched? *J. Clin. Med.* **9**, 879 (2020). 10.3390/jcm9030879 PMC7141526 32210197

[12] Shreve P, Chiao PC, Humes HD, Schwaiger M, Gross MD (1995). Carbon-11-acetate PET imaging in renal disease. J. Nucl. Med..

[13] Mikkelsen EFR (2017). Hyperpolarized [1-(13)C]-acetate renal metabolic clearance rate mapping. Sci. Rep..

[14] Leung, K. *Molecular Imaging and Contrast Agent Database (MICAD)*) (National Center for Biotechnology Information (US), 2004). 20641179

[15] Hosgood SA, Barlow AD, Hunter JP, Nicholson ML (2015). Ex vivo normothermic perfusion for quality assessment of marginal donor kidney transplants. Br. J. Surg..

[16] Dumbill, R. et al. Transplant and recipient factors in prediction of kidney transplant outcomes: a UK-wide paired analysis. *J. Clin. Med.* **11**, 2222 (2022). 10.3390/jcm11082222 PMC9024822 35456312

[17] Hui S (2020). Quantitative fluxomics of circulating metabolites. Cell Metab..

[18] Jang C (2019). Metabolite exchange between mammalian organs quantified in pigs. Cell Metab..

[19] Weissenbacher A (2019). Twenty-four-hour normothermic perfusion of discarded human kidneys with urine recirculation. Am. J. Transplant..

[20] Aburawi MM (2019). Synthetic hemoglobin-based oxygen carriers are an acceptable alternative for packed red blood cells in normothermic kidney perfusion. Am. J. Transplant..

[21] Zlatev H, von Horn C, Kaths M, Paul A, Minor T (2022). Clinical use of controlled oxygenated rewarming of kidney grafts prior to transplantation by ex vivo machine perfusion. A pilot study. Eur. J. Clin. Invest..

[22] Zager RA, Johnson AC, Becker K (2014). Renal cortical pyruvate depletion during AKI. J. Am. Soc. Nephrol..

[23] Arykbaeva AS (2021). Metabolic needs of the kidney graft undergoing normothermic machine perfusion. Kidney Int..

[24] Hoogland ER (2013). The value of machine perfusion biomarker concentration in DCD kidney transplantations. Transplantation.

[25] Kirchner C (2015). Ex vivo use of a Rho-kinase inhibitor during renal preservation improves graft function upon reperfusion. Cryobiology.

[26] Gracia-Sancho J (2010). Flow cessation triggers endothelial dysfunction during organ cold storage conditions: strategies for pharmacologic intervention. Transplantation.

[27] Alejandro V (1995). Mechanisms of filtration failure during postischemic injury of the human kidney. A study of the reperfused renal allograft. J. Clin. Invest..

[28] Legrand M, Mik EG, Johannes T, Payen D, Ince C (2008). Renal hypoxia and dysoxia after reperfusion of the ischemic kidney. Mol. Med..

[29] Leone G, Puliatti C, Morale W, Furnari M, Leone F (1995). [Warm ischemia in kidney from non-heart beating donor. Instrumental evaluation]. Minerva Chir..

[30] Demos DS (2015). Successful porcine renal transplantation after 60 minutes of donor warm ischemia: extracorporeal perfusion and thrombolytics. ASAIO j.

[31] Hosgood SA, Bagul A, Yang B, Nicholson ML (2008). The relative effects of warm and cold ischemic injury in an experimental model of nonheartbeating donor kidneys. Transplantation.

[32] Minor T, Sitzia M, Dombrowski F (2005). Kidney transplantation from NHBD after oxygenated low flow machine perfusion preservation with HTK. Transplant. Int..

[33] Thuillier R (2014). Cyclodextrin curcumin formulation improves outcome in a preclinical pig model of marginal kidney transplantation. Am. J. Transplant..

[34] Minor T, von Horn C (2019). Rewarming injury after cold preservation. Int. J. Mol. Sci..

[35] von Horn C (2023). The impact of oxygen supply and erythrocytes during normothermic kidney perfusion. Sci. Rep..

